# Impact of the LCBO strike on Ontario emergency department visits with an alcohol-related chief complaint: a natural experiment in alcohol policy

**DOI:** 10.3389/fpubh.2026.1730458

**Published:** 2026-03-02

**Authors:** Melissa Wan, Janice Mok, Angela Ma, Megan A. Carter, Allison Maier, Nancy Slipp, Adam van Dijk, Harmun Dhindsa, Daphne Mayer, Piotr Oglaza

**Affiliations:** 1 Department of Family Medicine, Queen's University, Kingston, ON, Canada; 2 Southeast Public Health, Kingston, ON, Canada; 3 Department of Public Health Sciences, Queen's University, Kingston, ON, Canada

**Keywords:** acute care use, alcohol availability, alcohol policy, natural experiment, poisson regression analysis, syndromic surveillance

## Abstract

**Background:**

Alcohol is a leading preventable cause of death and disability in Canada. Alcohol-related emergency department (ED) visits place a substantial burden on the health system, with evidence that greater alcohol availability contributes to higher utilization and related harms. The July 2024 Liquor Control Board of Ontario (LCBO) strike provided a natural experiment to evaluate the impact of reduced alcohol availability on healthcare utilization.

**Methods:**

An interrupted time series analysis was conducted to evaluate ED visits with an alcohol-related chief complaint, as captured in the Acute Care Enhanced Surveillance System for Ontario, across three time periods: before, during, and after the LCBO strike. Run charts were utilized to compare visit patterns with historical trends. Poisson regression models, both unadjusted and adjusted for weekly temporal patterns, were used to assess changes in visit rates.

**Results:**

ED visits with an alcohol-related chief complaint decreased by 24% during the LCBO closure (aRR: 0.76, 95% CI 0.66–0.88) and declined over the strike period (aRR: 0.98, 95% CI 0.97 – 1.00). They increased by 32% when LCBOs reopened (aRR: 1.32, 95% CI 1.02 – 1.70). Comparing 2024 7-day moving averages with 2023 values (± 1SD and ± 2SD) demonstrated that ED visits during the 2024 closure were significantly lower than in 2023.

**Interpretation:**

Reduced alcohol availability during the LCBO strike was associated with a significant decline in ED visits with an alcohol-related complaint, underscoring the potential health system benefits of reducing alcohol availability. The findings may inform policymakers considering measures to reduce alcohol-related healthcare burdens.

## Introduction

Alcohol is the most commonly used substance in Canada (1) and remains a leading preventable cause of death, disability, and social problems (2). Acute harms, including injuries, accidents, violence, and alcohol poisoning, frequently require emergency medical attention (3, 4). Chronic consumption is associated with liver disease, cardiovascular conditions, mental health disorders, and various cancers, often requiring long-term medical care (1, 2). In Ontario alone, alcohol was responsible for 6,202 deaths, 47,526 hospitalizations, and 258,677 emergency department (ED) visits in 2020, with total alcohol-attributable healthcare costs of $2.3 billion (4). The burden is also rising: between 2003 and 2016, ED visits attributable to alcohol increased four times more than all-cause ED visits (5).

Determinants of alcohol consumption are complex and influenced by government policies shaping availability and accessibility (6). Availability refers to the number, density, and operating hours of retail outlets, where consumption can be off-premise (e.g. liquor stores) or on-premise (e.g. bars and restaurants). Accessibility is shaped by age restrictions, income, cultural norms, and system-level factors such as pricing and enforcement of sales regulations (7). Canadian studies consistently show that greater alcohol availability, often through privatization and expanded retail access, is associated with higher consumption (8) and increased healthcare utilization (9–12). The effects are pronounced in lower socioeconomic areas, contributing to widening health inequities (11).

In Ontario, the Liquor Control Board of Ontario (LCBO) is the primary alcohol retailer, accounting for more than half of the volume of alcohol sales per year (13). In July 2024, a province-wide LCBO workers' strike led to the temporary closure of 680 LCBO stores. This disruption created a unique natural experiment to examine the effects of restricted alcohol availability on alcohol-related health system use (14).

The primary objective of this study was to leverage near real-time data from the Acute Care Enhanced Surveillance (ACES) System (15) to characterize and analyze trends in ED visits with chief complaints attributable to alcohol use before, during, and after the LCBO strike. This study examines whether reduced availability of off-site alcohol outlets is associated with changes in healthcare utilization.

## Methods

The study employed a natural experimental design using an interrupted time series analysis to study alcohol-related ED utilization before, during, and after the strike event.

The intervention period, characterized by an unplanned reduction in alcohol availability, spanned from July 05 to July 22, 2024, during which 680 LCBO stores across Ontario were closed (14, 16). Notably, certain alcohol outlets remained operational during this period, including private retailers, LCBO Convenience outlets, licensed grocery stores, The Beer Store, winery, cidery, and distillery outlets, along with bars and restaurants (14).

The control periods were the time immediately preceding the strike (May 05–July 04, 2024) and the time after the strike (July 23–Aug 31, 2024), and comparison data from the same time periods in 2023.

The analysis was guided by the hypothesis that changes in alcohol availability would result in immediate population-level effects on acute alcohol-related harms, consistent with known day-of-week patterns in alcohol use. Given the limited duration of the intervention, effects on chronic outcomes were not expected. Segmentation boundaries were therefore defined by the dates of LCBO closure and reopening.

### Data sources

The study used de-identified patient data from the ACES system (15). ACES is a syndromic surveillance system that monitors more than 99% of all provincial ED records; 97% of Ontario hospitals share data with ACES. It collects near real-time, de-identified patient data including date, time, age, sex, first five postal code digits, the Canadian Triage Acuity Score (CTAS), and a free-text reason for presentation. ACES uses natural language processing (NLP) to categorize words and phrases from the chief complaint or reason for admission into defined syndromes. The methods for the development of the NLP algorithms have been previously detailed (15, 17).

### Ethics approval

The data analyses were performed using anonymized, aggregate data in accordance with the ethical and legal limitations outlined in the data sharing agreements between ACES, public health agencies, and participating hospitals in Ontario and thus were exempt from Research Ethics Board review.

### Syndrome definition

The ACES system's alcohol (EOH) syndrome captures ED presentations related to alcohol use, including intoxication, addiction, withdrawal, and associated organ damage. In anticipation of the strike, the syndrome was expanded to improve surveillance of alcohol-related ED visits, including both consumption- and withdrawal-related visits. The enhanced definition included chief complaints containing “alc^*^” or “etoh” in addition to those classified under the pre-existing EOH syndrome.

Syndrome validation is conducted semi-regularly by comparing ACES counts to provincial acute care data from the National Ambulatory Care Reporting System (NACRS) held by the Canadian Institute for Health Information (CIHI). NACRS data serve as the “gold standard” for ED visit data, but these data are only available months after the patient visit (15). The linear correlation between ACES and CIHI data is plotted to ensure excellent correlation between the datasets. In 2021, the correlation between the EOH syndrome and the NACRS data was 0.804 with *p*-value < 0.001 (18), indicating strong alignment but suggestive of an underestimation of alcohol-related visits, likely due to limitations in chief complaint specificity and/or single-syndromes classification.

To support public health monitoring during the strike period, a daily surveillance report was generated using the enhanced EOH syndrome. This report included ED visit counts up to midnight of the previous day and was circulated to regional and provincial public health authorities. It provided baseline data from several weeks prior to the strike and historical comparisons from previous years, enabling contextual interpretation and timely response planning.

### Outcomes

The primary outcome of the study was the number of ED visits with alcohol-related chief complaints, as captured by the ACES system's enhanced EOH syndrome definition, aggregated by day for all of Ontario.

### Statistical analysis

Run charts were first created with daily counts, 7-day moving averages, and ±1 and ±2 standard deviations (SD) from the previous year's (2023) 7-day moving averages to visualize the data over time. Run charts are a typical tool used for situational awareness to identify unexpected increases or decreases in regularly collected epidemiological data (19). During July 2024, the run charts were calculated daily to monitor impacts of the strike on alcohol-related ED visits.

A crude and adjusted Poisson segmented regression model was fitted to the data using the generalized linear model with log link function in R (20–22). The base date was May 07, 2024, the close date July 05, 2024, and the reopen date was July 23, 2024. Dummy variables were created for when LCBO stores closed (*date LCBO closed*), when LCBO stores reopened (*date LCBO reopened*), and for each day of the week. The time variables calculated for inclusion in the model were *days since base date, days since LCBO closed*, and *days since LCBO reopened*.

Of interest was a level and slopes model, to understand not only the hypothesized drop and subsequent rebound in the number of ED visits, but also how the number of ED visits changed over time during the strike. The crude model includes the variable time and the dummy variables for when the LCBO was closed and reopened, and was specified as follows:

ln(*Y*_*t*_) = β_0_+β_1_ *dayssincebasedate*_*t*_+β_2_ *date LCBO closed*_*t*_+β_3_ *dayssinceLCBOclosed*_*t*_ +β_4_ *dateLCBOreopened*_*t*_+β_5_*dayssinceLCBOreopened*_*t*_+ *e*_*t*_

The adjusted model includes the dummy variables Tues through Sun and omits Mon as the reference to adjust for autocorrelation and seasonality (21). The adjusted model was checked for fit using Pearson's goodness of fit test and by examining the model's residuals graphically. Autocorrelation was checked using the autocorrelation and partial autocorrelation functions (23). Since the daily number of ED visits for the enhanced EOH syndrome were few, no adjustment was made for clustering of hospitals in the main model. This would have made a GEE or mix model unstable. Instead, a sensitivity analysis was conducted ad-hoc by aggregating hospitals to a region (with 5 regions) and included these regional dummy variables in the model as fixed effects.

The previous year, 2023, was selected for the run chart comparison because it is the year least likely to have been affected by the pandemic compared to 2020–2022. Initially at the time of writing, the 2025 May-Aug timeframe had not yet occurred. This was later added as a comparison year in the run chart analysis. Regional trends were examined observationally in 2022, 2023, and 2024.

## Results

**Figure 1:**
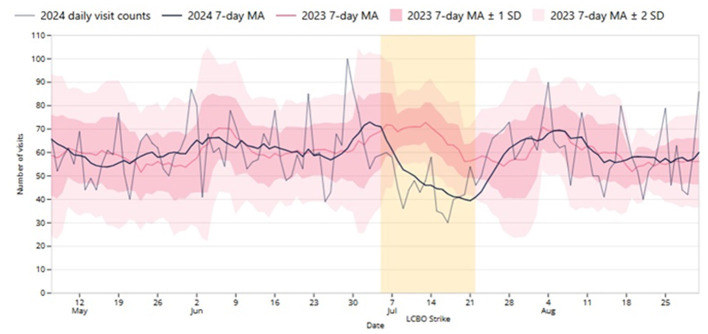
The daily number of emergency department (ED) visits classified as alcohol-related by the Acute Care Enhanced Surveillance (ACES) system's enhanced EOH syndrome from May 07 to August 31, 2024, with 7-day moving average (MA) shown for the study period (2024) and historical (2023) 7-day MA with both ± 1 (dark pink) and ± 2 (light pink) standard deviations (SD). The Liquor Control Board of Ontario (LCBO) strike period is highlighted in yellow (July 05 to July 22, 2024).

Figure 1 shows the daily and 7-day average ED visits for the enhanced EOH syndrome (i.e., with an alcohol-related chief complaint) in 2024. For comparison purposes, the 7-day average, and ± 1 and ± 2 SD from the same date in 2023 are shown. In the period before the strike (May 07–July 04), daily visits ranged between 39 and 100, with a daily average of 61.2. During the strike (July 05–July 22, highlighted in yellow), daily visits ranged from 30 to 60 and the daily average dropped to 45.6. Post-strike (July 23 to Aug 31), the minimum and maximum, were 40 and 90, respectively, and the average for daily visits rebounded to 61.1. In the same calendar periods for 2023, the averages for pre-strike, during strike, and post-strike periods were 60.7 (minimum 34, maximum 100), 64.8 (46, 81), and 59.0 (35, 78), respectively. The trend of the 2024 7-day moving average (bold blue line) was visually observed to change immediately following the strike and again at the completion of the strike. When compared with the 2023 7-day moving average (red line), the daily 2024 counts (light blue line) and the 2024 7-day average fell more than 2 SD below the 2023 7-day moving average (light pink area) during the strike period, suggesting a significant reduction in alcohol-related ED visits. The probability of the 7-day average being below 2 SD due to chance alone is extremely low at *p* < 0.00001. A similar, although slightly smaller difference was found with 2025 data where the 2024 7-day moving average was consistently below 1 SD and often, but not always below 2 SD.^1^

Table 1 details the adjusted Poisson model results. Key findings include significant reductions in ED visits with alcohol-related chief complaints as estimated by the enhanced EOH syndrome during the LCBO store closures (aRR 0.76, 95% CI 0.66–0.88, *p* < 0.001), and a slight downward trend over time during the closure (aRR 0.98, 95% CI 0.97–1.00, *p* < 0.01). Following the reopening, visits significantly increased (aRR 1.32, 95% CI 1.02–1.70, *p* < 0.05). Weekly patterns showed higher visit risks on Fridays (aRR 1.11, 95% CI 1.01–1.21, *p* < 0.05), Saturdays, and Sundays (both aRR 1.32, 95% CI 1.21–1.45, *p* < 0.001), compared to Mondays. Model checking demonstrated an adequate fit to the data with no evidence of autocorrelation.

**Table 1:** Adjusted Poisson model parameter estimates for emergency department (ED) visits classified as alcohol-related by the Acute Care Enhanced Surveillance (ACES) system's enhanced EOH syndrome during the Liquor Control Board of Ontario (LCBO) strike in July 2024.

**Parameter**	**Adjusted RR**	**95%CI**
Intercept	52.0	47.4–57.0^***^
**Days of the week (Mon** = **ref)**		
Tues	0.96	0.87–1.05
Wed	0.93	0.85–1.03
Thurs	1.04	0.95–1.14
Fri	1.11	1.01–1.21^*^
Sat	1.32	1.21–1.45^***^
Sun	1.32	1.21–1.45^***^
Days since base date	1.00	1.00–1.00^*^
Date LCBOs closed	0.76	0.66–0.88^***^
Days since LCBOs closed	0.98	0.97–1.00^**^
Date LCBOs reopened	1.32	1.02–1.70^*^
Days since LCBO reopened	1.01	1.00–1.02

^*^ < 0.05. ^**^ < 0.01. ^***^ < 0.001.

**Figure 2:**
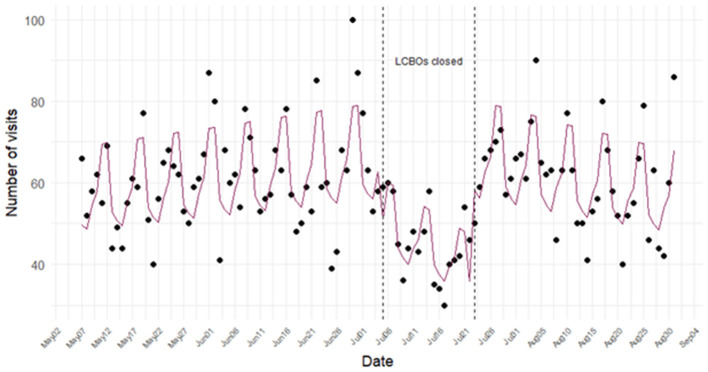
Poisson interrupted time-series fitted model (adjusted for day of the week) of the daily number of emergency department (ED) visits classified as alcohol-related by the Acute Care Enhanced (ACES) system's enhanced EOH syndrome before, during, and after the Liquor Control Board of Ontario (LCBO) strike in 2024.

Figure 2 shows the daily ED visits for alcohol-related complaints estimated by the enhanced EOH syndrome, adjusted for the day of the week. The decrease in visits during the LCBO store closures persisted after accounting for weekly variations, suggesting that the decline was not a result of the usual day-of-week patterns.

**Figure 3:**
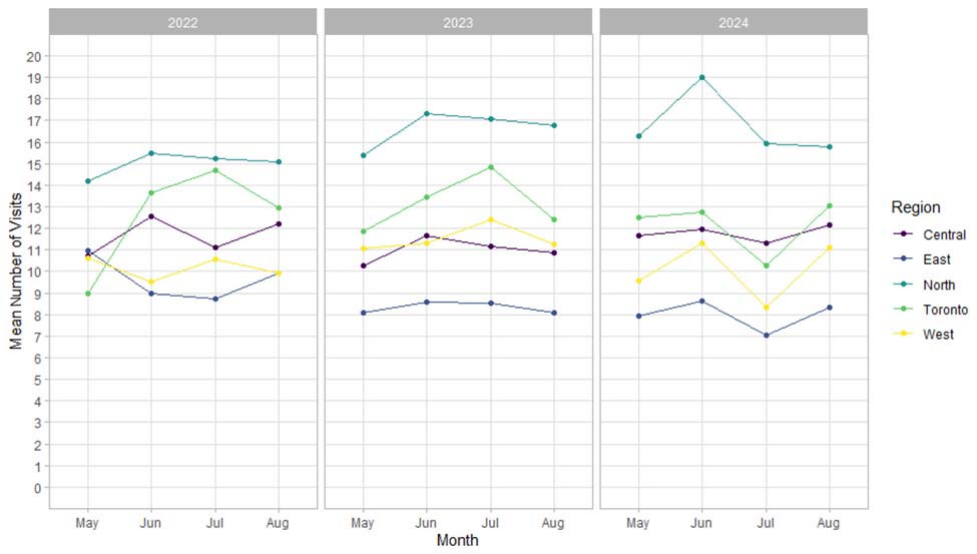
Sensitivity analysis showing mean monthly emergency department (ED) visits classified as alcohol-related by the Acute Care Enhanced Surveillance (ACES) system's enhanced EOH syndrome by Ontario region and year (2022–2024).

The addition of the regional fixed effects did not affect the model parameters (Table 2). As shown in Figure 3, average alcohol-related ED visit volumes varied across the regions, with higher levels observed in the north. Despite these baseline differences, the pattern observed in 2024 (a pronounced decline followed by a partial recovery) was evident across all regions. Comparable trajectories are not observed in 2022 or 2023.

**Table 2:** Sensitivity analysis including region as a fixed effect showing adjusted Poisson model parameter estimates for emergency department (ED) visits classified as alcohol-related by the Acute Care Enhanced Surveillance (ACES) system's enhanced EOH syndrome during the Liquor Control Board of Ontario (LCBO) strike in July 2024.

**Parameter**	**Adjusted RR**	**95%CI**
Intercept	10.4	9.4–11.9^***^
**Days of the week (Mon** = **ref)**		
Tues	0.96	0.87–1.05
Wed	0.93	0.85–1.03
Thurs	1.04	0.95–1.14
Fri	1.11	1.01–1.21^*^
Sat	1.32	1.21–1.45^***^
Sun	1.32	1.21–1.45^***^
**Region (central** = **ref)**		
East	0.68	0.62–0.74^***^
North	1.42	1.33–1.53^***^
Toronto	1.03	0.96–1.11
West	0.86	0.79–0.93^***^
Days since base date	1.00	1.00–1.00^*^
Date LCBOs closed	0.76	0.66–0.88^***^
Days since LCBOs closed	0.98	0.97–1.00^**^
Date LCBOs reopened	1.32	1.02–1.70^*^
Days since LCBO reopened	1.01	1.00–1.02

^*^ < 0.05. ^**^ < 0.01. ^***^ < 0.001.

### Interpretation

The 2024 LCBO strike raised concerns about changes in alcohol-related ED utilization. The disruption in alcohol availability was anticipated to influence a broad spectrum of alcohol-related presentations. The enhanced EOH syndrome was developed to improve detection of these visits, including both consumption- and withdrawal-related complaints (18). This study illustrates that ACES, combined with regression analysis, can rapidly identify potential public health impacts and support timely communication to decision-makers.

Study results show a significant 24% relative reduction in alcohol-related ED visits immediately upon store closures, with a modest continued decline throughout the strike. Run charts confirmed that this decline was statistically significant, as visits declined more than 2 SD below the 7-day moving average for the same calendar period in 2023. ED visits rebounded rapidly when LCBO stores reopened.

The sensitivity analysis introducing fixed effects did not impact the Poisson adjusted regression results (Table 2). As shown in Figure 3, four of the five regions exhibited similar trajectories in the average number of hospital visits from May to August in 2024, characterized by a clear decline from June to July followed by a rebound in August. The northern region differed, showing a pronounced decline from June to July without a subsequent rebound; this may reflect an unusually high baseline in that region relative to trends observed in 2022 and 2023. Although regional trajectories in 2022 and 2023 did not mirror those observed in 2024, absolute visit volumes remained fairly stable from year to year.

These findings align with prior evidence consistently linking policies that reduced availability of off-site alcohol retailers to decreases in alcohol consumption and related healthcare utilization (12, 24–27). For example, following Ontario's 2015 deregulation allowing alcohol sales in grocery stores, ED visits entirely attributable to alcohol increased by 6% in affected regions compared to regions without, with an overall rise of 24,000 visits from 2013/14 to 2016/17 (12). Another Ontario-based study found that each 10% increase in spatial accessibility score for off-site alcohol outlets between 2013 and 2019 was associated with a 4% increase in alcohol-related ED visits; regions in the highest decile of access experienced nearly 40% higher ED visit rate compared to those with the lowest access (26). This relationship persisted for people without a history of alcohol-related health-care use. An analysis focusing on the Northwestern Health Unit catchment found that an area experiencing the LCBO closures on specific days in 2022/2023 had significantly fewer all-cause and alcohol-related Ontario Provincial Police and Emergency Medical Services calls for service compared to an area that did not have the closure (27).

The present study is a unique natural experiment that captures the immediate response to a temporary decrease in alcohol availability, demonstrating an association with decreased alcohol-related healthcare utilization. Recent natural experiments of federal or regional policies to restrict alcohol availability have shown positive population health outcomes such as decreased alcohol consumption, decreased overweight/obesity, violence, and mortality rates (28, 29). Although these studies are not directly comparable to the current study due to differences in contexts (e.g., cultural, population, size) and scale of restrictions (e.g., temporary ban on all alcohol sales or complete prohibition of alcohol), they further bolster the evidence base for government policies to restrict, not increase, alcohol availability.

The results add to growing evidence that strategies targeting the physical and commercial environments in which alcohol is accessed are critical for reducing health and system burdens, rather than relying on targeted health education or other individual-level interventions (6, 7, 30, 31). Approaches such as limiting outlet density, setting minimum unit pricing, increasing minimum purchase age, and restricting marketing have been shown to reduce consumption and harms at the population level (6, 7, 30–32). In contrast, Ontario's recent expansion of alcohol sales to 8,500 private retailers—a 289% increase—raises concerns about additional strain on an already overburdened healthcare system (33).

Future research should examine both short- and long-term impacts of alcohol control measures, not only on population per capita consumption or individual health outcomes, but also on broader societal and economic indicators. Natural experiments and quasi-experimental methods remain important for this work, particularly when paired with economic evaluations that capture direct healthcare and social costs (e.g., policing and correctional services), which can inform prioritization of certain policies or interventions (34).

There is the possibility that a health communication campaign, an event that kept everyone at home, or some other population-based intervention to reduce unhealthy drinking can explain the results seen. However, the authors are unaware of such interventions or events, which would have had to occur province wide. This is also less likely, given how abruptly the LCBO stores closed and the simultaneous and significant drop in alcohol-related ED visits. Given that this is an ecological-level study, it is unknown the degree to which smaller geographical areas are affected by the strike. However, observationally, it appears that this trend occurred across the province in the broad regions examined.

The present study uses data from the ACES system which collects more than 99% of provincial ED registration records, yet, as a syndromic system based on triage text, may not capture all alcohol-attributable ED visits. Given the general performance of ACES in capturing alcohol-related visits as evidenced by strong agreement with CIHI's ambulatory data (*r* = 0.804) (18), it is likely that the trends observed here are accurate, but the number of visits is underestimated, thus limiting effect-size estimation; this is a critical component for quantifying the impact of these policies. Therefore, these findings should be validated using CIHI's ambulatory data holdings to examine visits 100% attributable to alcohol.

It should be noted that some off-site retailers remained operational during the LCBO strike, and that the impact observed may be underestimated.

Overall, this study suggests that reduced alcohol availability due to the LCBO strike was associated with a significant decline in ED visits with alcohol-related chief complaints. These findings add to the evidence linking reduced physical availability of off-site alcohol retailers with lower healthcare system impacts from alcohol consumption. The study also highlights the value of real-time syndromic surveillance of ED presentations, which can help identify both expected and unexpected health effects of external events, and support timely public health responses.

## Data Availability

The data analyzed in this study is subject to the following licenses/restrictions: Data is restricted to decision-makers and data analysts working in the Ontario public health system Requests to access these datasets should be directed to https://www.kflaphi.ca/contact-us/.

## Footnotes

1 ACES did not receive hospital records from the Chatham-Windsor area from October 2023 to March 2025. This would not affect results as ED visits with an alcohol-related chief complaint in July 2023 or 2025 were less than 1.3% of Ontario visits in the same periods.
